# The whole is more than the sum of the parts: establishing an enabling health system environment for reducing acute child malnutrition in a rural South African district

**DOI:** 10.1093/heapol/czz060

**Published:** 2019-07-06

**Authors:** Helen Schneider, Maria van der Merwe, Beauty Marutla, Joseph Cupido, Shuaib Kauchali

**Affiliations:** 1 School of Public Health and SAMRC Health Services to Systems Unit, University of the Western Cape, Robert Sobukwe Road, Bellville, South Africa; 2 Mpumalanga Department of Health, No 7 Government Boulevard, Mbombela, South Africa; 3 National Department of Health, Civitas Building, 222, Thabo Sehume St, Pretoria, South Africa

**Keywords:** Enabling environment, acute malnutrition, nutrition, district, health system strengthening, story of change, childhood mortality

## Abstract

There is a gap in understanding of how national commitments to child nutrition are translated into sub-national implementation. This article is a mixed methods case study of a rural South African health district which achieved accelerated declines in morbidity and mortality from severe acute malnutrition (SAM) in young children, following a district health system strengthening (HSS) initiative centred on real-time death reporting, analysis and response. Drawing on routine audit data, the declining trends in under-five admissions and in-hospital mortality for SAM over a 5-year period are presented, comparing the district with two others in the same province. Adapting Gillespie *et al.*’s typology of ‘enabling environments’ for Maternal and Child Nutrition, and based on 41 in-depth interviews and a follow-up workshop, the article then presents an analysis of how an enabling local health system environment for maternal-child health was established, creating the conditions for achievement of the SAM outcomes. Embedded in supportive policy and processes at national and provincial levels, the district HSS interventions and the manner in which they were implemented produced three kinds of system-level change: knowledge and use of evidence by providers and managers (‘ways of thinking’), leadership, participation and coordination (‘ways of governing’) and inputs and capacity (‘ways of resourcing’). These processes mainstreamed responsibility, deepened accountability and triggered new service delivery and organizational practices and mindsets. The article concludes that it is possible to foster enabling district environments for the prevention and management of acute malnutrition, emphasizing the multilevel and simultaneous nature of system actions, where action on system ‘software’ complements the ‘hardware’ of HSS interventions, and where the whole is more than the sum of the parts.

**Key Messages**

- There is a gap in understanding of how national commitments to child nutrition are translated into sub-national implementation.
- Building enabling environments at local or district level is key to implementation of nutrition interventions.
- Based on a case study of positive change, this article provides insights into the elements of an enabling local health system environment for addressing acute child malnutrition in South Africa.

## Introduction

The evidence on what is required to prevent and address child malnutrition is well-established. A Lancet Series on Maternal and Child Nutrition (*The Lancet*, 2013) spelt this out as a combination of ‘nutrition-specific’ and ‘nutrition-sensitive’ interventions and programmes. The nutrition-specific interventions include, amongst others, breastfeeding, dietary and micronutrient supplementation and treatment of severe acute malnutrition (SAM). Nutrition-sensitive approaches are the actions in health and other sectors that enable good nutrition, such as food security, early childhood development, water and sanitation, families and women’s education and empowerment, social protection and accessible health services (*The Lancet*, 2013).

In summarizing the state of the field, the Lancet Series pointed to an important gap in knowledge, namely, on how to secure the implementation of nutrition programmes at scale in real-world settings, and called for more research on how to shape and sustain enabling environments for nutrition. Elaborating on this, Gillespie *et al.* (2013, p. 553) defined enabling environments as ‘the political and policy processes that build and sustain momentum for the effective implementation of actions that reduce undernutrition.’ They further proposed a typology of enabling environments, categorized into (1) knowledge and evidence; (2) politics and governance; and (3) leadership, capacity and financial resources. These ‘drivers’ can be viewed along pathways of change from commitment to implementation and impact (Gillespie *et al.*, 2013).

This theory-based approach to the analysis of drivers and pathways of nutrition was further elaborated in the Story of Change (SoC) initiative (Gillespie and van den Bold, 2017). Using a common methodology and framework, comprehensive case studies were conducted of countries or regions within countries that have achieved some success in confronting high levels of childhood stunting.

From these and other case studies, there is a growing understanding, firstly, of the social and economic conditions underpinning nutrition improvements, and secondly, of the factors which generate national commitment to nutrition (Pelletier *et al.*, 2012; Webb *et al.*, 2016; Harris *et al.*, 2017; Baker *et al.*, 2018). Global initiatives such as Scaling up Nutrition and the Decade of Nutrition have placed nutrition firmly on national political and policy agendas. However, there is as yet insufficient understanding of how political attention translates into budgetary commitments and co-ordinated action through health and other sectors from national to sub-national and local levels (Pelletier *et al.*, 2012).

Malnutrition remains a significant problem in South Africa, despite the country’s status as an upper-middle-income country, general declines in under-five mortality over the last decade and successes in the prevention of mother-to-child transmission of HIV (Bamford *et al.*, 2018). In 2016, more than a quarter (27.4%) of children under 5 years of age were stunted (low height-for-age), and more than one-in-ten (13.3%) had low weight-for-height (National Department of Health *et al.*, 2017). SAM is a direct or contributory cause to 31% of deaths in children under 5 years, while a further 27% of deaths are associated with milder forms of undernutrition (Bamford *et al.*, 2018).

South Africa has scored consistently high on global indices of national commitment to addressing nutrition, such as the Hunger and Nutrition Commitment Index for Africa (africa.hancindex.org/countries/south-africa). The country has an overarching National Policy on Food and Nutrition Security (Departments of Social Development and Agriculture Food and Fisheries, 2013), a multisectoral National Food and Nutrition Security Plan (2017–2022) (Department of Planning Monitoring and Evaluation, 2017), and a health sector Roadmap for Nutrition in South Africa a systematic, evidence-based framework seeking to ‘reposition nutrition and nutrition related issues and actions prominently in the health care system’ (National Department of Health, 2013, p. 8). However, as has been described in other contexts, national policy does not guarantee sub-national implementation (Pelletier *et al.*, 2012). A key challenge in implementing nutrition-related interventions in South Africa’s quasi-federal governance system is the co-ordination of actors—horizontally across sectors and vertically across spheres of government.

Even within the health sector, sustaining change at the coal-face of service delivery is not straight forward. Interventions in training and support of health workers over a decade in two district hospitals of one province, Eastern Cape, achieved short-term reductions in SAM mortality but these were poorly sustained over time (Ashworth *et al.*, 2004; Puoane *et al.*, 2008; Muzigaba *et al.*, 2017). Emphasizing the complexity of the challenge, the authors of these interventions concluded on the need for quality improvement (QI) strategies combined with broader health systems, including team work and leadership, strengthening.

This article is a case study of an enabling environment created for nutrition-specific interventions in a rural South African district, within a context of active provincial support and a national policy framework. The district achieved accelerated declines in hospital admissions and in-patient mortality from SAM in young children over a 4-year period. The article first describes the changes in SAM outcomes in the district and then analyses how a more general health system strengthening (HSS) initiative established an enabling environment for the SAM outcomes. The article adds to the literature on how national commitments are translated into sub-national implementation, and extends understanding on building capacity for addressing nutrition and other health priorities at the frontline of health systems.

## Methods

### The setting

Gert Sibande District is one of three districts in Mpumalanga Province of South Africa, and has a population of 1.1 million (Massyn *et al.*, 2017). Typical of predominantly rural provinces, Mpumalanga has higher levels of poverty and unemployment and lower educational levels and access to sanitation and electricity, than the rest of South Africa (Table 1). The province is situated in the east of the country, where the HIV epidemic is most concentrated, and had an estimated population HIV prevalence of 13.0% in 2015, compared with 11.3% for South Africa as a whole (Day and Gray, 2017). Antenatal HIV prevalence was 35% in 2015 (National Department of Health, 2017).

**Table 1:** Demographic, socio-economic and health profile

	South Africa	Mpumalanga Province
2018 population (million)	55.9	4.4
Unemployment rate (%)	26.5	31.0
Households experiencing food adequacy (%)	78.7	69.1
Poverty prevalence (%)	54.4	62.4
Female literacy +20 years (%)	93.1	85.8
Improved water source (%)	92.5	91.4
Improved sanitation (%)	79.9	65.8
Public health sector dependent population (%)	84.2	87.1
Life expectancy at birth (years)	62.4	55.8
HIV prevalence 2015 (ASSA)%	11.3	13.0

*Sources:* Massyn *et al.* (2017), Day and Gray (2017) and StatsSA (2018).

The three districts in the province—Gert Sibande, Ehlanzeni and Nkangala—although with distinct histories, share similar socio-economic profiles. However, Nkangala District, being more inland, had a lower estimated HIV prevalence of 11% in 2016, compared with 17% in the other two districts (Massyn *et al.*, 2017).

As with the rest of the country, the public sector is by far the most significant provider of health services in the districts. Gert Sibande provides primary health care (PHC) services, free at the point of use, through a network of 85 clinics and community health centres. These refer to eight district hospitals, and one regional hospital. The district is led by a District Manager who is supported by a District Health Management Team, with some functions delegated to hospital and PHC managers in seven sub-districts.

In a 2014 national review of progress towards achievement of the Millennium Development Goals (MDG), Gert Sibande was identified as one of several districts with above average maternal and under five mortality. In particular, the case fatality from SAM was 28%, the highest in the country. From 2014 onwards, two facilitators were contracted to provide mentorship and support to the district in improving their maternal, neonatal and child health (MNCH) outcomes. While focusing on specific problems such as SAM, the approach of the facilitators was on strengthening the local health system to better respond to district MNCH priorities.

### Conceptual framework, data collection and analysis

The analysis draws on Gillespie *et al.*’s (2013) typology of enabling environments examined along the pathways of change from commitment, to implementation and impact. These drivers of change in nutrition are adapted for a district (rather than national) context and for impacts on acute rather than chronic malnutrition. Following the SoC approach (Gillespie and van den Bold, 2017), the methodology combines a quantitative analysis of changes in morbidity and mortality, drawing on routine data from Mpumalanga Province, and a qualitative assessment based on interviews and a workshop with a wide cross section of district, provincial and national players.

#### Quantitative data and analysis

Provincial data on SAM admissions and deaths under 5 years of age were extracted from the Child Healthcare Problem Identification Programme (ChIP), a national mortality audit system in existence for over 10 years. This is regarded as the most authoritative source of data on SAM in the Province. Other indicators (pneumonia and diarrhoea) and population estimates were extracted from the provincial routine district health information system (DHIS) for the 5-year period 2013/14–2016/17. National level indicators and prevention of mother-to-child transmission of HIV (PMTCT) data were obtained from the Health System Trust’s database of health indicators (www.hst.org.za/healthindicators). The HST database compiles information from the DHIS and periodic surveys.

Table 2 lists the morbidity and mortality indicators reported. Trends in quantitative data are presented by district in Mpumalanga for a 5-year period starting prior to the district interventions (financial years 2013/14–2017/18).

**Table 2:** Morbidity and mortality indicators

Indicator	Numerator	Denominator	Period
Rate of under-5 hospital admissions for SAM	Hospital admissions for SAM under-5 years	1000 under 5-population	2013/14–2017/18
Rate of under-5 in-hospital SAM mortality	In-hospital deaths from SAM	10 000 under-5 population	
SAM case fatality rates	In-hospital SAM deaths under-5 years	Hospital admissions for SAM under-5 years	
Percentage decline in SAM admissions and deaths under-5 years	Hospital admissions and deaths from SAM under-5 years 2014/5 minus 2017/18	Hospital admissions and deaths for SAM under-5 years 2014/15	2014/15^a^–2017/18
Rate of under-5 hospital admission for pneumonia	Hospital admissions for pneumonia under-5 years	1000 under-5 population	2013/14–2017/18
Rate of under-5 hospital admission for dehydrating diarrhoea	Hospital admissions for diarrhoea with dehydration under-5 years	1000 under-5 population	
Prevention of mother to child transmission of HIV rates (percentage)	HIV PCR positive tests at around 6 weeks	All HIV PCR tests done at around 6 weeks	2011/12–2015/16

a Period starting with the peak levels of mortality.

#### Qualitative data and analysis

The qualitative component of the study was undertaken in a sequence of steps as follows:

Step 1: During the course of 2017, an independent research team conducted 41 interviews with a cross section of players in Gert Sibande District, as part of a wider evaluation of the HSS initiative in four districts. Of the seven sub-districts in Gert Sibande, two were purposefully selected as representing different degrees of adoption and buy-in to the new strategies (high and low). Interviewees included district and sub-district line managers, dietitians, programme managers, frontline providers from hospital, primary health care and community services, support staff and partners (Table 3). ‘Partners’ included the main facilitator, a HIV/AIDS non-governmental organization, and a university academic co-ordinating service learning in the District.

**Table 3:** Distribution of interviewees

Category	*n*
Level	
Community services	2
Sub-district and PHC	6
Hospital	19
District	14
Total	41
Professional category	
District and hospital dietitians	4
District MCH programme managers and technical experts	7
Line managers: district, sub-district, facility and community services	17
Other (EMS, information managers, social services)	10
Partners	3
Total	41

EMS, Emergency Medical Services; MCH, maternal-child health.

Interviews were guided by a semi-structured interview schedule developed on the basis of a document review and interviews with the facilitators of the system strengthening initiative. The interviews combined considerations of context, intervention design, actors, co-ordination and roll-out processes, values and principles, framing and communication strategies and outcomes.

The interviews were recorded, transcribed, coded and analysed thematically by a team of four researchers. Following the case study methodology (Yin, 2014), the individual sub-districts were analysed separately and then combined with each other and the district-level analysis. The research team met on a regular basis, iteratively developing the key conclusions of the study. The findings were documented in a draft technical report and validated in a feedback workshop held in the district after which the report was finalized (Schneider *et al*., 2017).

Step 2: On the basis of the wider evaluation, we identified the response to acute malnutrition in Gert Sibande District as an embedded case (Yin, 2014) for further evaluation. In April 2018, the research team convened a follow-up workshop with 19 representatives from the national and provincial health departments, and key players from the district. The purpose was to specifically explore the changes in the District’s response to acute child malnutrition and agree on the key elements of the further analysis reported in this article. An authorship team consisting of both insiders and outsiders to the district and the nutrition interventions was constituted. This team identified the SoC methodology as the best overall approach to the case study, sourced additional routine data, constructed timelines for the nutrition interventions and revisited the interview data.

**Figure 1:**
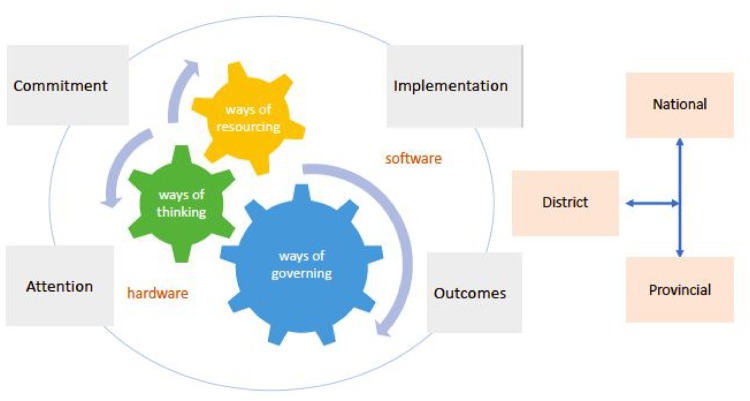
Analytic framework of an enabling district environment for nutrition.

Step 3: Based on the co-authors’ joint reflections and consensus developed over the course of several months, an adapted version of Gillespie *et al.*’s (2013) analytic framework of an enabling environment was developed for presenting the findings, summarized in Figure 1. This represents the enabling environment as a dynamic across stages of the policy process, influenced by actors within the district and beyond, involving both system ‘hardware’ and ‘software’ (Sheikh *et al.*, 2011), and producing three kinds of system-level change: ways of thinking, ways of governing and ways of resourcing (described further in Table 4).

**Table 4:** Typology of enabling environment

	Gillespie *et al.* (2013)	Adapted domains
Drivers of change	Framing, generation and communication of knowledge and evidence	Ways of thinking: knowledge, framing and use of evidence
	Political economy of stakeholders, ideas and interests	Ways of governing: leadership, participation and co-ordinated action
	Capacity (individual, organizational, systemic) and financial resources	Ways of resourcing: inputs and capacity
Pathways of change	Commitment to implementation	Commitment to implementation

The overall evaluation proposal was approved in a briefing meeting with senior district players, and signed consent was obtained for all the in-depth interviews.

## Findings

### Timeline and key activities

Supplementary File S1 provides a timeline of the key provincial and district activities.

Training courses on the management of SAM for district clinicians were first convened by the Mpumalanga Provincial nutrition programme from late 2013 onwards. In 2014, district clinical specialist teams (DCSTs) were appointed to support MNCH services, and in 2015, intensified provincial processes of nutrition planning, support, monitoring and procurement of essential supplies were set in motion.

Against this back drop of provincial support, HSS interventions were initiated in Gert Sibande District from late 2014 onwards. An experienced mentor, who had played a leadership role in district development in another province, was appointed to support the District in improving its MNCH outcomes. With national, provincial and district backing, he established district and sub-district meetings referred to as ‘Monitoring and Response Units’ (MRUs), and a system of ‘real-time’ (24 h) death reporting, analysis and response. The participants in the MRUs included line, support and clinical staff from hospitals, PHC and community services; partners (e.g. academic, NGO) based in the district; and the South African Social Security Agency (SASSA) which administers the government system of child care grants and food distribution to families in distress.

The monthly MRU meetings focused on analysing maternal, under five and neonatal deaths in hospitals, and developing co-ordinated responses to prevent future deaths at all levels. SAM became a particular focus of the MRU. In a linked set of activities, a second facilitator designed and implemented a QI initiative in PHC clinics, centred on enhanced information use, QI tools (targets, run charts) and participatory problem-solving (improvement cycles). The work of the two facilitators was supplemented by occasional direct support to individual facilities from the National Department of Health and other technical experts. All the interventions worked with the available resources and systems of the district and province, and apart from the part-time presence of the two facilitators (who were also supporting other districts in the country), no additional external funding was mobilized.

These interventions were widely understood to have prompted changes in the delivery of maternal and child health services, including in the approach to child malnutrition. This was described by one of the dietitians as follows: ‘We have been fighting SAM for a very long time. I remember the days when … we were riding donkey carts. Now we are flying in aeroplanes.’

With respect to SAM, the first change was a high level of adherence to the ‘WHO Ten Steps’ for management of SAM (Ashworth *et al.*, 2003) in district hospitals. ‘In casualty they were trained… they know that from the ten steps they have to feed immediately…. From casualty, they’ll admit immediately in the ward so that they implement all those steps… without even waiting for the dietitian (hospital dietitian)’.

Functional systems of referral and continuity of care were established between hospitals, clinics and community outreach teams as well as with SASSA. There was overall much greater awareness of, and attention to, early detection of malnutrition in the district as a whole. ‘Most of the time we don’t wake up being a SAM. Which means this child has been missed at a certain level and we are the culprit, at primary health care, be it at a mobile, be it at a clinic, because this child has been seen there…. So through the programme, we’re able to train them, able to make them vigilant, to identify these children so that they are able to be sent as quickly as possible…’ (PHC manager).

### Quantitative indicators

**Figure 2:**
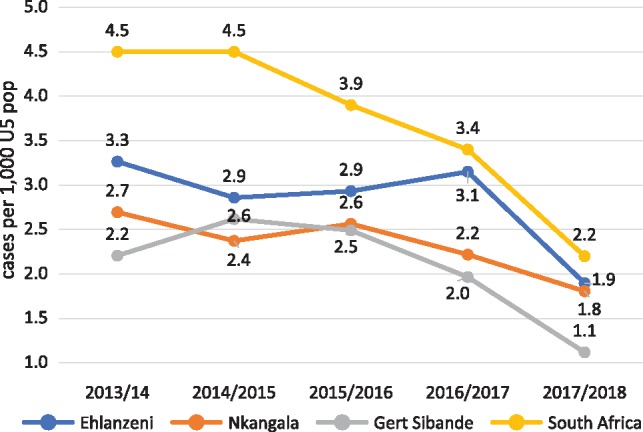
Trends in hospital admissions for SAM per 1000 under-five population in Mpumalanga districts (compared with South Africa), 2013/14–2017/18 (*Source*: ChIP for Mpumalanga, DHIS for South Africa).

**Figure 3:**
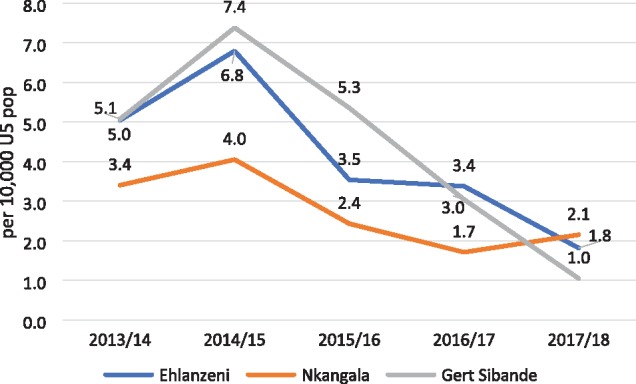
Trends in hospital deaths from SAM per 10 000 under-five population in Mpumalanga districts, 2013/14–2017/18 (*Source*: ChIP data).

**Figure 4:**
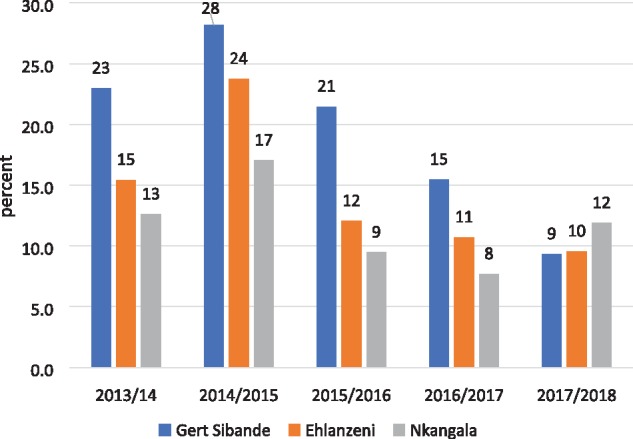
Trends in SAM facility case fatality rates in Mpumalanga districts (2013/14–2017/18) (*Source*: ChIP data).

**Figure 5:**
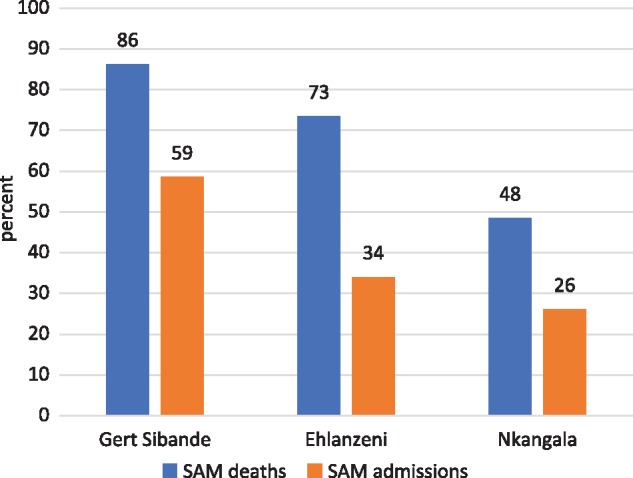
Percentage decline in SAM hospital admissions and in-patient deaths in Mpumalanga districts (from peak mortality levels in 2014/15–2017/18) (*Source*: ChIP data).

As with the rest of South Africa, all three districts in Mpumalanga Province recorded significant declines in hospital admissions for SAM over the 5-year period (2013/14–2017/18) (Figure 2). The declines in Gert Sibande District were more pronounced and began earlier than the other two districts, starting in the year after interventions began (2015/16). In-patient deaths from SAM peaked in 2014/15 (Figure 3), reflected in the high case fatality rates in that year (Figure 4). In-patient death and case fatality rates declined steeply thereafter, again most noticeably in Gert Sibande District. The percentage decline in both admissions and in-patient deaths was greatest in Gert Sibande District (Figure 5).

Similar trends of declining admissions for pneumonia and diarrhoea and mother-to-child transmission of HIV were recorded over the 5-year period in Mpumalanga Province (presented in Supplementary File S2). These have been accompanied by improvements in coverage of antenatal care <20 weeks, Vitamin A, and exclusive breastfeeding of young infants (Day and Gray, 2017).

### Enabling environment and pathways of change

In themselves, the nutrition-specific and system strengthening interventions described above are not unusual, and not the first of such initiatives in the District and the Province. Indeed, while this set of interventions was implemented in one district, an observation during the evaluation was the large number of other parallel QI and audit processes (some of them long-standing) targeting all three districts. It is important therefore, to understand *how* the apparently straight forward interventions implemented in Gert Sibande District were able to plausibly achieve better outcomes.

Based on the enabling environment typology, this section explores the underlying drivers and pathways of change in Gert Sibande District, categorized as: ways of thinking, ways of governing and ways of resourcing, along a pathway from initial district engagement and buy-in (commitment) to implementation and impact.

#### Ways of thinking

Ways of thinking relate to how issues and problems are framed, the evidence that is marshalled and the manner in which this is communicated to system actors, such that it draws their attention and commitment.

The global MDG Countdown to 2015 processes (Bhutta *et al.*, 2010), in which South Africa was portrayed as under-performing on mortality goals, (re)-established MNCH as a national priority. Through analysis of routine health information system data, a handful of districts, among them Gert Sibande, were identified as high-mortality areas holding back the country’s progress. This had a powerful galvanizing effect on frontline actors. As recounted by a PHC supervisor in one of the districts: ‘we were shown the letter that was from Dr [senior national manager] that was actually showing that the national is not happy about us, because we had a lot of maternal and child death caused by …SAM, malnutrition and diarrhoea. So after that, then we had to go to the core of what is it that’s not going right in our district’. Reducing MNC mortality, and in particular, responding better to malnutrition, became constructed as a priority for senior and mid-level district managers, and laid the ground for entry and support from the facilitators.

Simultaneous to this district ‘naming and shaming’, national expert committees were collating and packaging the global and national evidence-base into structured and simplified guidance for frontline health system players. Nine key focal areas to reduce MNC mortality were proposed, one of which was child nutrition. The national Road Map for Nutrition further concretized the approach to malnutrition, including endorsing food supplementation and the ‘WHO Ten Steps’ (Ashworth *et al.*, 2003) for the management of SAM. These national policy and evidence-based guidelines provided the necessary templates for district actors to organize responses.

The new approaches to monitoring and information use introduced through MRUs enabled district actors to prioritize which interventions to implement. The 24 h death reporting of maternal, perinatal and child deaths to designated district programme and clinical managers was at the heart of this, provoking regular interactions between district and frontline players. Structured analysis of deaths (into avoidable, modifiable and unavoidable causes) formed the starting point of discussions in the MRUs. Through root cause analysis, hospital-level mortality was further linked to failures at PHC and community levels. MNCH mortality (‘survive’) were linked to service coverage (‘thrive’) indicators, and reported in standard dashboards (example in Supplementary File S2).

By connecting individual indicators into broader patterns, formal information was rendered meaningful. ‘It made sense when Dr [facilitator] was explaining the reasoning behind the information … I think that is one of his roles, to make you understand that this is not just numbers, these numbers are saying something’ (district dietitian).

It also provoked further enquiry and dialogue. ‘We sit together and then they say, okay: “Breastfeeding, what happened in the hospital, what happened in the clinic?” So everyone is answering. It was reported on paper but it wasn’t verbalised. So by verbalising now you can immediately say “oh sister you say this, but what about that?” Because now you are starting to engage and I think that makes the difference… it is interactive’ (paediatric ward nurse manager).

With time, improved information use extended beyond the MRU and became integrated into routine monthly and quarterly reviews at facility, sub-district and district level. ‘We are looking at data in a different manner. We look at what happened, using the “four R’s”. The first R is we record, the second R we report, the third R we review and the fourth R we respond’ (PHC supervisor).

A key framing device which promoted systems thinking was the use of the ‘open tap’ analogy, a public health metaphor which links prevention and early treatment (turn off the tap) with preventing deaths (plug the hole). The ‘open tap’ was frequently invoked in interviews: ‘We are using that open tap analogy everywhere now… I like it because it clearly describes that to say, as long as … you haven’t identified the root causes and make sure you don’t temporarily put a block, but close the tap’ (PHC manager). Similarly, slogans such as ‘no child with SAM will walk alone’ symbolized the child as accompanied across levels of care and sectors in a seamless continuity of care; the ‘four R’s’ represented the need to ‘close the loop’ by linking death audits with responses.

These various framing, analytic and sense-making strategies helped to shift attitudes to malnutrition, which became viewed as abnormal or unacceptable. ‘One death [from SAM] is too many. So we need to avoid that so that it doesn’t become a habit that people will just die (hospital dietitian)’.

In sum, national policy, evidence-based guidelines and local child mortality audits played an important role in creating district-wide attention and commitment to addressing malnutrition. Equally important was how this formal knowledge combined with informal, tacit and experiential knowledge and imagery, thereby enabling the mindset shifts and systems thinking required for new forms of collaborative service delivery.

#### Ways of governing

Governance refers to direction, oversight and accountability, as well as how roles and responsibilities are allocated between actors. The manner in which national policy cascaded to provincial level planning, followed by implementation, and the regular communication between these levels, provided a vital context for the response to malnutrition in Gert Sibande District.

Against this enabling background, the facilitators in the District sought to strengthen existing district oversight mechanisms (such as PHC review meetings) while establishing MRUs as a new co-ordination and accountability structure specifically targeting MNCH outcomes.

The district and sub-district MRUs had a deliberate design. They were constituted of representatives from the ‘management triangle’—referred to as drivers, navigators and experts. The drivers were the line managers (district manager, hospital CEO, PHC manager) who convened meetings and held other actors accountable; the navigators were the information managers and public health specialists who monitor progress and support planning; and the experts were the clinicians (specialist medical and nursing staff, dietitians) who provide training on, and technical support for the key interventions. Each set of actors was portrayed as having a unique and essential role in the district response, with shared responsibility for MNCH, and within this SAM. In this way, nutrition became ‘everybody’s business’: ‘Everyone is playing his or her role, that’s what I can say… we are working like this, because the one can’t survive without the other’ (hospital CEO). This promoted respectful engagement: ‘If I have to give advice to another district, it is work with this commitment, with this respectful way, to acknowledge the capacity, the potential of the other colleagues’ (district clinician).

While the district manager (at district level) and hospital chief executive officer (at sub-district level) were formally in charge of meetings, in practice leadership of MRU activities was distributed. Informal alliances, referred to by the project facilitator as the ‘individuals or groups who welcome innovation and change for quality improvement’ were encouraged. Dietitians, who previously had been more peripheral players, emerged as key leaders of the response to SAM at a sub-district level. They were able to mobilize, train and oversee a range of other actors over whom they did not necessarily have line authority. ‘I also did the SAM training, like with night staff and paeds, [and] I am training the clinics on when actually to refer’ (hospital dietitian).

MRU meetings had structured agendas and a specific focus. Clarity of expectations enabled action in complex systems with multiple problems: ‘that thing of you sitting there wondering what actually do they want now. But now there is a template and they are specific about what they want’ (PHC manager). Meetings were regarded as effective and tasks were taken seriously. ‘You know sometimes you will attend a lot of meetings where we will talk and come back and talk about the same thing next month. But there is no implementation and there is no accountability. So if you attend a meeting where people are serious and they are taking the task that is given seriously and they come back and report, then I think it is worth to attend’ (hospital dietitian).

A key feature of the MRU design was the way it connected players—in particular, community, PHC and hospital actors. Improved vertical relationships were repeatedly raised as a major achievement of the MRUs: ‘the difference that I am seeing is that there is a very good relationship between the hospitals and the local clinics, because now they communicate, they meet together’ (district programme manager).

These improved relationships also included new forms of team work within facilities and wards. ‘It’s like the doctor would come in the ward, the sister would do the ward rounds with the doctor alone without involving the social worker, the dietitian, the physio, the OT [occupational therapist]… But now it has improved a lot. They are working as a team’ (district dietitian).

Systems of informal communication were also greatly enhanced. ‘I know when I have a problem in the paediatric ward, quickly I need to pick up the phone and call primary health care to say I have admitted a child who is like this and this…’ (hospital nursing manager).

Although initially resisted as an ‘add-on’, the shared leadership and accountability, the new relationships forged and the empowerment of actors, accompanied by tangible successes, established the MRU as a favoured mechanism of district and sub-district governance. ‘You know, deaths, fewer deaths is very powerful. And clearly it’s connected [to MRU]’ (hospital CEO); ‘They never thought that as a team they can do more. So now because they are seeing a lot of changes… the team work is even stronger’ (district dietitian); ‘The MRU has brought about ownership and accountability on the part of the managers and also the health professionals at facility level’ (district programme manager).

#### Ways of resourcing

A key factor in the successes of the MRU in Gert Sibande District was how expectations of greater accountability were matched by new forms of support for providers. ‘I think people were hiding some of whatever is happening, but now people are open because they see that there is support’ (district programme manager). Enhanced support included new systems of cascaded training (including on SAM), targeted mentoring of individual facilities or sub-districts, and improved procurement and continuity of supplies, especially food supplementation.

As indicated earlier, these additional resources were mobilized from within the system. The provincial, and to some extent the national programme, played an important role in ensuring the availability of essential supplies and organizing training. The inter-sectoral relationship established with SASSA added further tangible resources. Within the district, existing staff were deployed in ways that strengthened organizational capacity. Specialist clinicians (allocated to the district office and in the regional hospital) as well as programme managers (MNCH and nutrition) managed the 24 h death reporting system, visited facilities, distributed guidelines and provided day-to-day mentoring. In 2016, a newly appointed District Director added the MRU as a core mandate in the performance agreements of all line managers. By spreading responsibility, the strategies were less vulnerable to the loss of capacity caused by a high turnover of frontline providers or individual champions. Further, stewardship of the response to malnutrition was placed with dietitians, a relatively stable professional group compared with other clinicians (in particular doctors, who are often seen as the ‘natural’ team leaders).

Table 5 summarizes the elements of the enabling environment for addressing acute child malnutrition in Gert Sibande District.

**Table 5:** Drivers and pathways of change in Gert Sibande District (software elements indicated in brackets)

Drivers	Attention and commitment	Implementation
Ways of thinking: framing and use of evidence	MDG countdown influence Naming and shaming high burden districts (software) Priority interventions identified Guidelines available	Real-time death reviews Routine data analysis Use of tacit knowledge (software) Metaphors and slogans (software)
Ways of governing: leadership, participation and co-ordination	National policy Provincial plans Provincial support and commitment District commitment Involvement of SASSA	MRU as a governance structure connecting hospital, PHC, community; management triangle Increased, reciprocal accountability (software) Informal alliances (software) Empowered role of dietitians (software) Integrated into MNCH programme
Ways of resourcing: capacity and inputs	Improved provincial supply chains Appointment of DCSTs Mainstreaming responsibility	Training, supplies, ongoing mentoring Dietitians as stable players Linking audit with responses (‘4Rs’)

## Discussion

In the face of significant overall secular trends and in the absence of counterfactuals, it is not possible to attribute the declines in malnutrition admissions and mortality in Gert Sibande District solely to the system strengthening interventions described in this article. It is plausible that:

- the successes of the PMTCT programme, combined with declining diarrhoea and pneumonia incidence had a significant impact on SAM incidence in all three districts of Mpumalanga Province;
- similarly, intensified provincial support and the resulting improved management of SAM in hospitals led to the declines in case fatality rates; across the Province
- the additional SAM prevention and care interventions in Gert Sibande accounted for the earlier and steeper declines in SAM mortality and admissions in this District.

This case study offers insights into enabling environments for implementation of nutrition-specific interventions in district health systems. The SoC methodology (Gillespie and van den Bold, 2017) and the adapted version of the enabling environment typology (Gillespie *et al.*, 2013) provided coherent approaches for analysing how enabling environments are created.

A number of lessons emerge from the experiences of Gert Sibande District.

Firstly, it is possible to foster enabling health district environments for nutrition-specific interventions by mobilizing existing resources, mainstreaming nutrition into maternal-child health programmes, and placing ultimate responsibility for nutrition with sub-district and facility managers. This shifts the focus from models of clinician training and mentoring, previously implemented in South Africa (Ashworth *et al.*, 2004; Puoane *et al.*, 2008; Muzigaba *et al.*, 2017), to action at higher levels of the system and with a wider range of actors (Doherty *et al.*, 2018). System-level strengthening requires approaches which incorporate, but go beyond the technical evidence-base on nutrition, which include processes that:

- shape collective thinking through knowledge, evidence, framing and communication;
- pay attention to the allocation and distribution of roles and responsibilities, and establish accountabilities;
- build both individual and organizational capacity; and provide necessary support.

Secondly, successful district nutrition programmes are embedded in supportive relationships and processes at higher—in this instance, provincial and national—levels, providing policy, guidelines, material resources and technical expertise. ‘Whole system’ approaches, co-ordinating action at multiple levels, build enabling environments at the frontline (Samuels *et al.*, 2017).

Thirdly, pathways of change from commitment to implementation and impacts are not linear. Bureaucratic commitment by senior district managers may secure a degree of compliance but not necessarily buy-in at all levels of the system. In the face of multiple competing demands, mid-level managers and frontline providers do not necessarily see the need for new structures and processes. Shifts from grudging compliance of top-down instructions towards internalized commitment to implementation take time. They occur when successes are visible, actors are empowered, and when accountability is understood as reciprocal, namely, expectations of improved performance are matched with equal degrees of support. ‘Stages of change’ or pathways approaches (Belizán *et al.*, 2011) are helpful heuristics for assessing implementation but need to be understood ‘within a non-linear and dynamic model of change’ (Baker *et al.*, 2018).

Fourthly, local health systems have to be approached as fundamentally social systems, shaped by human agency and relationships. Successful strategies operate at two levels: the formal and informal; the official and unofficial; system hardware and software (Sheikh *et al.*, 2011). In the case study district, improved use of formal information occurred simultaneously with the promotion of informal knowledge through metaphors and symbols. Insistence on adherence to guidelines was matched with opportunities to elicit local experiential and tacit knowledge for problem-solving. The official system of governance in the MRU had its counterpart in unofficial ‘informal alliances’ of like-minded, public interested individuals; formal training with everyday support and mentoring. As pointed out ‘human and organizational capacity need to encompass not only nutrition know-how, but also a set of soft-power skills to operate effectively across boundaries and disciplines’ (Gillespie *et al.*, 2013, p. 554). The capacity to communicate is obviously a key element of this. This tactical know-how may reside less with technical nutrition or clinical experts than with skilled health system managers who have the knowledge and experience of navigating complex systems.

### Limitations

This case study has a number of limitations. The analysis is based on one case, and while the findings may be analytically generalizable, their validity would need to be tested in other settings (both positive and negative). The evaluation was also a retrospective one, in which events were reconstructed based in part on documentary evidence and in part on the accounts of interviewees, subject to recall bias. While the evaluation established agreement on key activities and their timing, it was not possible to develop a more granular account of specific factors and moments of change. A prospective design, with an audit trail of interventions and change at sub-district level would be required for this.

The quantitative findings are based on the analysis of routine data from health facilities, which are not generalizable to populations, and which vary in quality from facility to facility.

An enabling environment for addressing acute malnutrition is different to that required to address the persistent problem of childhood stunting and the growing problem of child obesity (Perez-Escamilla *et al.*, 2018). Both problems of under- and over-nutrition have their roots in the basic causes of very high levels of inequality in income and access to land, employment and other opportunities in South Africa (Devereux and Waidler, 2017). Health sectors can play a catalytic role in nutrition programmes but ultimately need support from other sectors and broader political commitment (Huicho *et al.*, 2016; Hossain *et al.*, 2017).

## Conclusions

This article has described the nature and mechanisms of a sub-national enabling environment for addressing acute child malnutrition in South Africa. The theory-based approach to drivers and pathways provides a coherent frame for examining local enabling environments. This analysis adds to existing frameworks by presenting change as a non-linear dynamic, by making explicit the interplay of system software and hardware, and by emphasizing the multi-level and simultaneous nature of system actions required, in which the whole is more than the sum of the parts.

## Ethical approval

The research protocol received ethical clearance from the University of Western Cape’s Biomedical Research Ethics Committee (#BM17_1_35), and was approved by the Mpumalanga Provincial Research Committee (#MP_2017RP50_441).

## Supplementary Material

czz060_Supplementary_Data
